# Supplementary material for: p21+TREM2+ senescent macrophages fuel inflammaging and metabolic dysfunction-associated steatotic liver disease
Source: Nat Aging. 2026 Apr 16;6(4):792–815. doi: 10.1038/s43587-026-01101-6 (PMC13099426; doi:10.1038/s43587-026-01101-6)
Supplement: Supplementary file 1 — Reporting Summary [file 43587_2026_1101_MOESM1_ESM.pdf]

nature portfolio

Corresponding author(s): Anthony J. Covarrubias
Last updated by author(s): Feb 19, 2026

Reporting Summary

Nature Portfolio wishes to improve the reproducibility of the work that we publish. This form provides structure for consistency and transparency in reporting. For further information on Nature Portfolio policies, see our Editorial Policies and the Editorial Policy Checklist.

Statistics

For all statistical analyses, confirm that the following items are present in the figure legend, table legend, main text, or Methods section.

n/a	Confirmed	
☐	☒	The exact sample size (n) for each experimental group/condition, given as a discrete number and unit of measurement
☐	☒	A statement on whether measurements were taken from distinct samples or whether the same sample was measured repeatedly
☐	☒	The statistical test(s) used AND whether they are one- or two-sided<br>*Only common tests should be described solely by name; describe more complex techniques in the Methods section.*
☐	☒	A description of all covariates tested
☐	☒	A description of any assumptions or corrections, such as tests of normality and adjustment for multiple comparisons
☐	☒	A full description of the statistical parameters including central tendency (e.g. means) or other basic estimates (e.g. regression coefficient) AND variation (e.g. standard deviation) or associated estimates of uncertainty (e.g. confidence intervals)
☐	☒	For null hypothesis testing, the test statistic (e.g. F, t, r) with confidence intervals, effect sizes, degrees of freedom and P value noted<br>*Give P values as exact values whenever suitable.*
☒	☐	For Bayesian analysis, information on the choice of priors and Markov chain Monte Carlo settings
☐	☒	For hierarchical and complex designs, identification of the appropriate level for tests and full reporting of outcomes
☐	☒	Estimates of effect sizes (e.g. Cohen's d, Pearson's r), indicating how they were calculated

Our web collection on statistics for biologists contains articles on many of the points above.

Software and code

Policy information about availability of computer code

Data collection	Bio Rad CFX MAESTRO software 3.1, Attune NxT software v3.1.2, ZEISS 3.11, -xcalibur 4.0
Data analysis	Image J 150i (western blot and microscopy), FlowJo v 10.1 (flow cytometry), R v4.5, RStudio 2025.05.1+513 (statistics and data science), Python is 3.13.5(data science and algorithms), Bio Rad CFX MAESTRO software 3.1(RT-qPCR), ZEISS 3.11 (microscopy). QuPath v0.6.0 (quantification). DESeq2 (v1.48.2).

For manuscripts utilizing custom algorithms or software that are central to the research but not yet described in published literature, software must be made available to editors and reviewers. We strongly encourage code deposition in a community repository (e.g. GitHub). See the Nature Portfolio guidelines for submitting code & software for further information.

Data

Policy information about availability of data

All manuscripts must include a data availability statement. This statement should provide the following information, where applicable:

- Accession codes, unique identifiers, or web links for publicly available datasets
- A description of any restrictions on data availability
- For clinical datasets or third party data, please ensure that the statement adheres to our policy

Raw SASPomics data and complete MS data sets were uploaded to the Center for Computational Mass Spectrometry, to the MassIVE repository at UCSD under the dataset identifier MSV000098058. The proteomics and metabolomics datasets generated and analyzed during this study have been deposited in the MassIVE

repository under the dataset identifier MSV000098413. All transcriptomic data, both Fastq and counts data, are publicly available for download in the Gene Expression Omnibus (GEO) database under the following accession numbers: Figure 1 (GSE318801), Figure 2 (GSE318651, GSE318804), Figure 4 (GSE318802), Figure 7 (GSE319035, GSE318652), and Figure 8 (GSE318806). Raw and normalized lipidomics data are included as supplementary data, source data are provided, and all other data supporting the findings are available from the corresponding author upon request.
Tabula muris sensis data set is publically accessible using the Chan Zuckerberg GitHub repository : czbiohub-sf/tabula-muris-senis

Research involving human participants, their data, or biological material

Policy information about studies with human participants or human data. See also policy information about sex, gender (identity/presentation), and sexual orientation and race, ethnicity and racism.

Reporting on sex and gender	NA
Reporting on race, ethnicity, or other socially relevant groupings	NA
Population characteristics	Peripheral blood mononuclear cells (PBMCs) were purified from the blood of anonymous healthy donors provided by UCLA's Virology core and research center. Per request we specified healthy males and females adults. The study only involved the use of human biological samples, obtained from the UCLA Virology and research center (Los Angeles, CA) and did not contain personal information about the identity, sex, or age of the donor and is therefore exempt from IRB approval.
Recruitment	Samples form human blood donors remained anonymous per UCLA's Virology Core and research center, Los Angeles, CA.
Ethics oversight	NA

Note that full information on the approval of the study protocol must also be provided in the manuscript.

Field-specific reporting

Please select the one below that is the best fit for your research. If you are not sure, read the appropriate sections before making your selection.

☒ Life sciences ☐ Behavioural & social sciences ☐ Ecological, evolutionary & environmental sciences

For a reference copy of the document with all sections, see nature.com/documents/nr-reporting-summary-flat.pdf

Life sciences study design

All studies must disclose on these points even when the disclosure is negative.

Sample size	The sample size and statistical analyses were determined prior to data collection. Sample sizes were chosen based on mouse availability and experimental feasibility. No statistical methods were used to pre-determine sample sizes for in vitro and in vivo studies. No data were excluded from the analyses. All in vitro experiments used at least 3 biological replicates to meet the minimum requirement for parametric statistical testing. In vitro experiments for transcriptomics, proteomics, metabolomics and SASPomics required at least 4-6 biological replicates were determined by sample availability and sequencing efficiency. The number of mice for each in vivo experiment was determined by the mouse availability. Power analysis was not performed to determine cohort size, instead the size was determined by animal availability and mouse number used in prior similar studies with expected similar effect size. In figure 3a, 8 aged C57BL/6J male mice (22-24 months) was collected based of animal availability and 8 young mice were used to match the aged mice cohort size. In figure 3b, 2 old (19 month) male livers were used based on liver tissue availability and 2 young (3 month) male livers were used to match the aged mice cohort. In figure 3c, representative images of one mouse per age, old male (24 months) and young male (3 months) was used based on tissue availability for this qualitative study. For in vivo experiments in figure 6(F-K) an N=12 C57BL/6J male mice (24 months) was determined by animal availability. Six aged mice were allocated per experimental condition (veh vs ABT). One vehicle control mouse died therefore the final vehicle control in N=5. N-6 young male mice was determined to match the aged mice cohort size. Figure 7 (A-E) in vivo experiments that involved HFHCD the CTEP APOE mutant mice, 25 male mice/strain (8-10 weeks old) were used. Figure 7(G-S) 20 CTEP APOE mutant male mice crossed to C57BL/6J mice were generated across 3 cohorts. An even number of mice were allocated per condition as a vehicle control or treated with ABT-263. Extended Data Figure 1(I-M), 6-10 male young (3 months) and old (26 months) CD38 KO mice used for in vivo gene expression experiments also determined by cohort availability. The experiments were not randomized. Investigators were not blinded to allocation during experiments and outcome assessment. Statistical analyses were performed using the latest Prism (v10.6.1) and R (v4.5.2). The specific statistical tests used for each experiment are described in the figure legends. All tests were two-sided unless otherwise stated, and a p-value < 0.05 was considered statistically significant. Data distribution was assumed to be normal, but this was not formally tested. Individual data points are shown where appropriate to illustrate the distribution and variability of the data. All experiments were repeated independently three times with similar results. Detailed protocols and analysis methods are provided to ensure reproducibility.
Data exclusions	No data exclusions were preformed.
Replication	All experiments presented in this manuscript was independently repeated 2-3 times and all attempts replicate findings made in this manuscript.

Randomization	The experiments were not randomized
Blinding	Blinding was not preformed because the experiments were not impacted by investigator bias.

Reporting for specific materials, systems and methods

We require information from authors about some types of materials, experimental systems and methods used in many studies. Here, indicate whether each material, system or method listed is relevant to your study. If you are not sure if a list item applies to your research, read the appropriate section before selecting a response.

Materials & experimental systems

n/a	Involved in the study
☐	☒ Antibodies
☐	☒ Eukaryotic cell lines
☒	☐ Palaeontology and archaeology
☐	☒ Animals and other organisms
☒	☐ Clinical data
☒	☐ Dual use research of concern
☒	☐ Plants

Methods

n/a	Involved in the study
☒	☐ ChIP-seq
☐	☒ Flow cytometry
☒	☐ MRI-based neuroimaging

Antibodies

Antibodies used

Target--Company--Catalog Number--Anti-(R=rabbit, r=rat)--Application
mouse beta-Tubulin--Cell signaling--2146S--R--western- dilution 1:1000
mouse Lamin B1--Cell signaling--13435T--R--western-dilution 1:1000
mouse P-Histone--H2AX--Cell signaling-- 9718T-- R--western-dilution 1:1000
mouse P-NF-kappaB p65--Cell signaling-- 3033T-- R--western-dilution 1:1000
mouse NF-kappaB p65--Cell signaling -- 8242T --R--western-dilution 1:1000
mouse p21 --Abcam --ab 188224-- R--western-dilution 1:1000
mouse TREM2-- Invitrogen --MA5-28223 --rat--western-dilution 1:1000
mouse CMPK2-- Abcam -- ab139720 -- R -- western-dilution 1:1000
mouse p16 -- Abcam -- 211542 - western-dilution 1:1000
mouse Tom20 -- proteintech -- 11802-1-AP- Immunofluorescence-dilution 1:1000
mouse dsDNA -- Abcam -- ab27156-1002 -- Immunofluorescence-dilution 1:1000
mosue F4/80 -- Abcam -- ab6640--rat -- Immunofluorescence-dilution 1:200
mouse p21 --Abcam --ab 188224-- R-- Immunofluorescence-dilution 1:200

Validation

All antibodies for flow cytometry applications were validated with proper isotype controls using primary mouse or human macrophages. Western blot antibodies, specifically mouse p21, p16, Trem2, and Cmpk2, were further validated by detecting bands of the correct size and when possible using samples from knockout mice that lack the target protein. The p21 Abcam antibody listed above was specific to p21 protein by western and for immunofluorescence. P16 was also tested in a similar method, however no commercially available antibody passed internal validations using whole body p16 KO mice for immunofluorescence. Therefore p16 antibodies were used only for western blot analysis, validated on BMDMs from mice lacking p16 in myelolid cells (LysMCre x p16 fl/fl). There are no commercial validated antibodies for the mouse Cmpk2 antibody, therefore, CRISPR was used disrupt the Cmpk2 locus and the Abcam antibody was specific to the Cmpk2 protein (please see Figure 2m). Our Trem2 antibody (Invitrogen MA5-28223) was tested against BMDMs from a Trem2 KO background and we found the Invitrogen antibody to be specific (please see figure 4f).

Eukaryotic cell lines

Policy information about cell lines and Sex and Gender in Research

Cell line source(s)	L929 cells used to make conditioned media to grow BMDMs were obtained from ATCC
Authentication	Cells were authenticated by ATCC. L929 cells were further validated by the ability of the supernatant rom these cells to differentiate and grow mouse BMDMs.
Mycoplasma contamination	L929 stocks kept in the laboratory were analyzed for Mycoplasma contamination annually and tested negative.

Commonly misidentified lines (See ICLAC register)	NA

Animals and other research organisms

Policy information about studies involving animals; ARRIVE guidelines recommended for reporting animal research, and Sex and Gender in Research

Laboratory animals	The following laboratory animals were used in vitro experiments. Briefly, 8-12 week male mice were sacrificed for bone marrow harvest then subjected to macrophage differentiation in vitro in the presence of mCSF. In figure 1-2; N=3 male C57BL/6J mice were used to perform experiments testing macrophage senescence. No power analysis was performed to pre-determine an N=3; this number was predetermined based on animal availability. Other B6 mice were used for In vivo testing for figure 3. In figure 3, N=16 male C57BL/6J mice was determined the cohort size based on animal availability. The experiment evaluated the expression of Cdkn1a, SA-Beta gal, and Immunofluorescence. Half the mouse cohort is young (2-4 months) the other half is old (22-24 months); N=8 mice per age group was used for statistical testing. Four heart samples (2 young, 2 old) were allocated for other experiments unrelated to this manuscript. In figure 4 C57BL/6J and Trem2 -/- mice were used to harvest bone-marrow-derived macrophages. N=6 C57BL/6J young (8-12 week) male mice were used for proteomic analysis (Figure 4a-c) based on animal availability. N=3 C57BL/6J (8-12 week) male mice was used as wild-type control for Trem2 -/- N=3 (8-12 week) male mice (figure 4f-j). N=3 male young (8-12 week) C57BL/6J was used to harvest bone-marrow-derived macrophages for lipidomics experiments and Ac-LDL in vitro experiments (figure 5a-c and figure 6a-e).<br><br>For in vivo studies the following mice were used. C57BL/6J and Cd38KO mice were from The Jackson Laboratory (Bar Harbor, ME cat#: 000664 & 003727). All transgenic mice expressing human CETP were obtained from The Jackson Laboratory, and mice carrying the human APOE*3-Leiden variants were kindly provided by Dr. L. Havekes. All mice were young male adults (2-4 months) or old males (22-24 months) and the genetic backgrounds include C57BL/6J, Cd38 KO, CETP-APOE mutant, BXD19, and 129X1. Mice were housed and maintained at UCLA animal facilities on a 12 hour dark/light cycle with ambient temperatures of 20-26C and the relative humidity of 30-70%. In figure 6f-k, N=23 C57BL/6J male mice (N=12 young and N=11 old) were predetermined sample size based on animal availability. N=6 mice were allocated for every group except for the old vehicle group where one mouse acquired the tumor. No power analysis was performed to determine the sample size. In figure 7a-d the generation and characterization of the F1 mice for this study were determined based on animal availability. N=25 three month CETP-APOE mutant, BXD19, and 129X1 mice were placed on a high fat high cholesterol diet to assess the impact of excess cholesterol on macrophage function and senescence in vivo. N=3-4 mice were harvested per time point and per genotype based on animal availability. In figures 7g-s, N=19 CETP-APOEx C57BL/6J male mice were used for senolytic testing. Sample size was not predetermined strictly due to animal availability. N=9 male mice were placed on a HFHCD and treated with Vehicle, N=10 male mice were given ABT-263.
Wild animals	No wild animals were used in this study.
Reporting on sex	All mice used for in vitro and in vivo studies were male.
Field-collected samples	NA
Ethics oversight	Mice were bred at UCLA in accordance with the Institutional Animal Care and Use Committee (IACUC). Animal studies were approved by the UCLA Animal Research Committee and IACUC.

Note that full information on the approval of the study protocol must also be provided in the manuscript.

Plants

Seed stocks	NA
Novel plant genotypes	NA
Authentication	NA

Flow Cytometry

Plots

Confirm that:

☒ The axis labels state the marker and fluorochrome used (e.g. CD4-FITC).

☒ The axis scales are clearly visible. Include numbers along axes only for bottom left plot of group (a 'group' is an analysis of identical markers).

☒ All plots are contour plots with outliers or pseudocolor plots.

☒ A numerical value for number of cells or percentage (with statistics) is provided.

Methodology

Sample preparation	All flow cytometry experiments were performed on in vitro macrophage cultures. Activated BMDMs or senescent BMDMs were lifted from non-TC treated plates after activation using cold PBS with 5mM EDTA for 5-10 min prior to blocking with FC block and staining with antibodies. Click-iT Edu 488 labeling (ThermoFisher) was used to measure macrophage proliferation as per manufacturer's protocol. Surface staining with other fluorochrome conjugated antibodies were performed for 30 minutes on ice at 1 ug for every mL each in PBS. Cells were then given an antibody safe fix solution, permeabilized, then subjected to intracellular staining on ice for 30 min in PBS. Cells were then moved for analysis on when the Attune NxT was performed.
Instrument	Attune NxT Acoustic Focusing Cytometer
Software	Attune NxT software v3.1.2 with quantification on FlowJo v10
Cell population abundance	All flow cytometry experiments were done in vitro using BMDM macrophage cultures. Cell abundance was determined by the number of macrophages positive for FFCH and SSCA.
Gating strategy	Viable macrophage populations in vitro were determined using FSC/SSC at approximately FAC 400k/SSC 150-600K based on voltage 80 FSC and 310 SSC.<br><br>Single cells were determined looking at FSC-H and FSC-A of a height to area ratio of one to one 1:1.

☐ Tick this box to confirm that a figure exemplifying the gating strategy is provided in the Supplementary Information.
