# Supplementary figures and images for: p21+TREM2+ senescent macrophages fuel inflammaging and metabolic dysfunction-associated steatotic liver disease
Source: Nat Aging. 2026 Apr 16;6(4):792–815. doi: 10.1038/s43587-026-01101-6 (PMC13099426; doi:10.1038/s43587-026-01101-6)

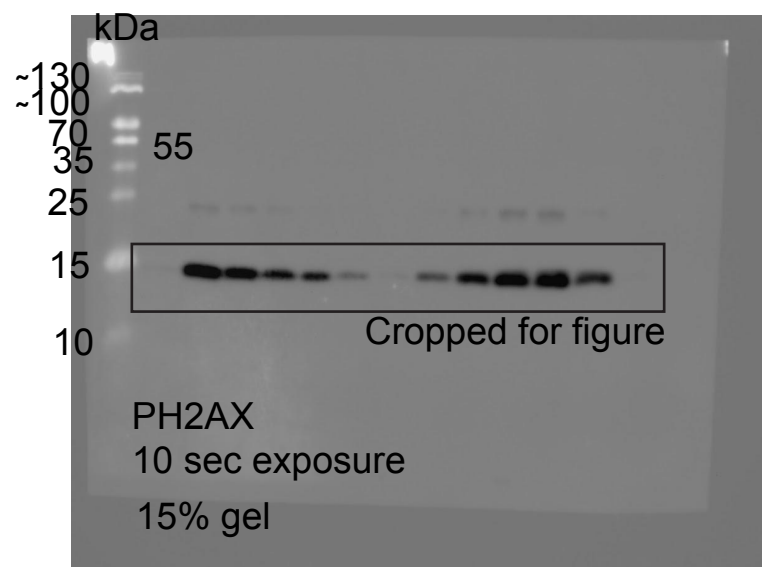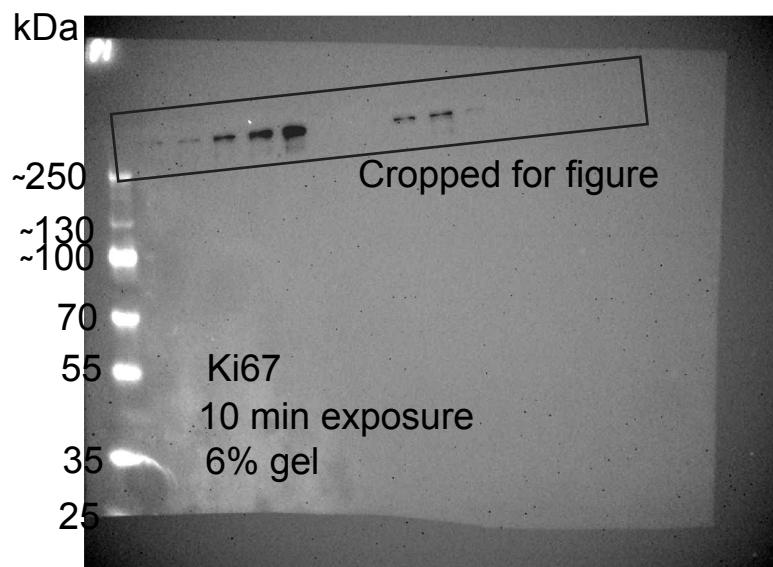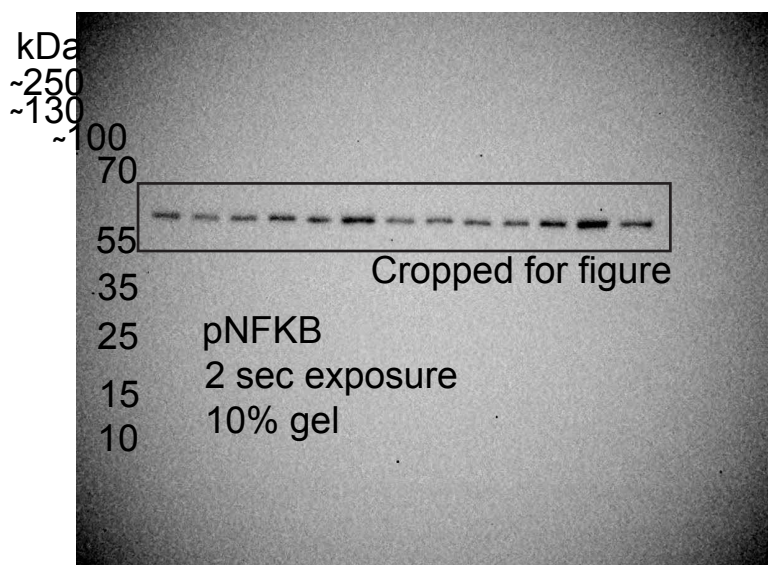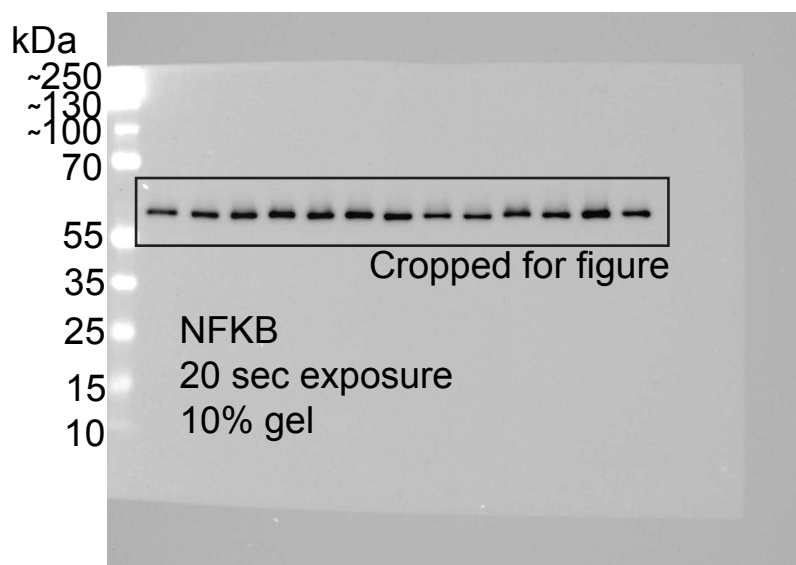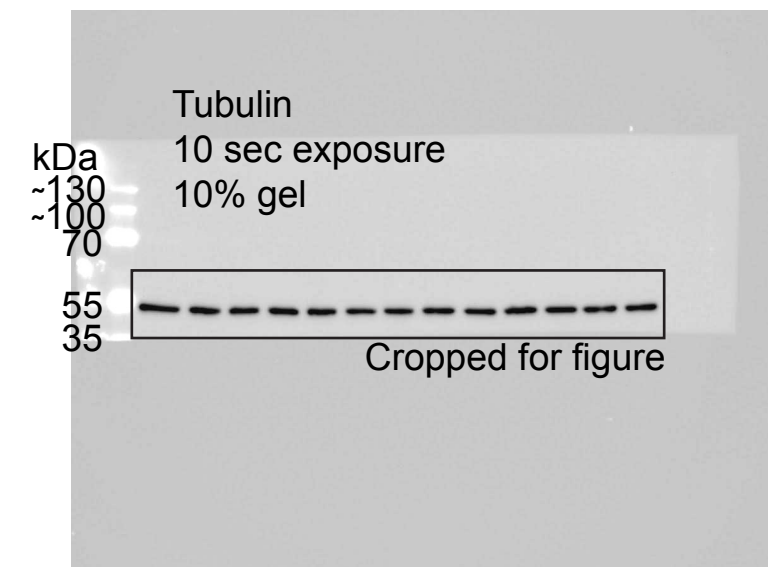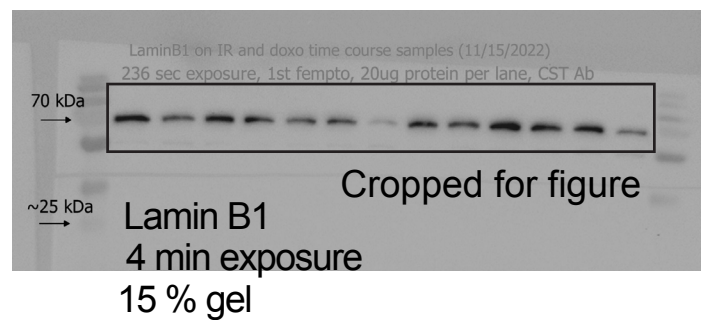

Supplement: Supplementary file 6 — Unprocessed western blots. [file 43587_2026_1101_MOESM6_ESM.pdf]

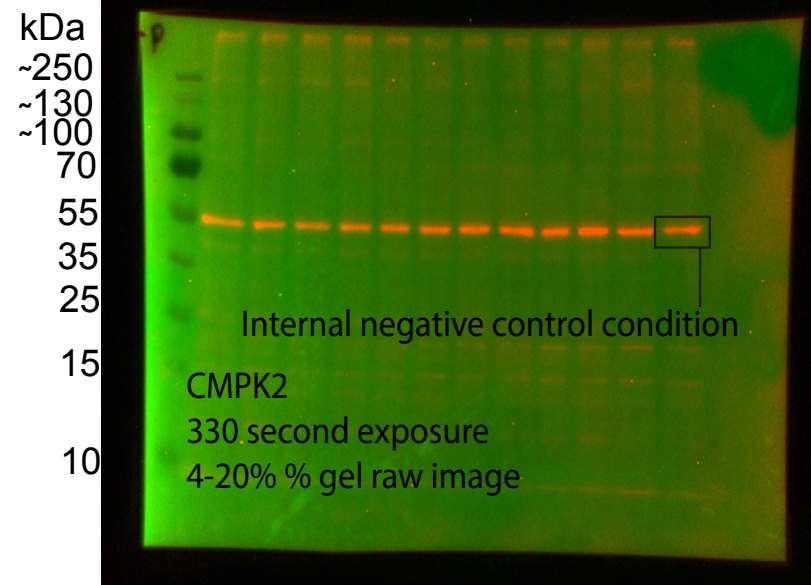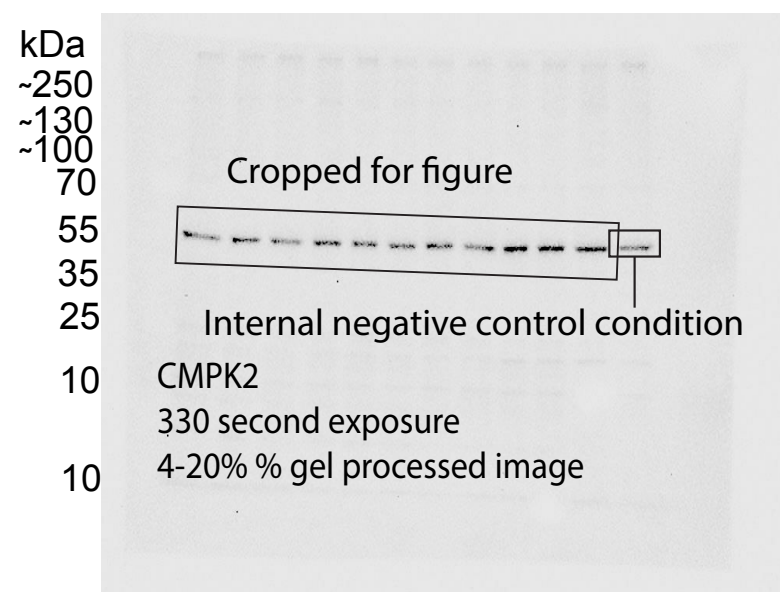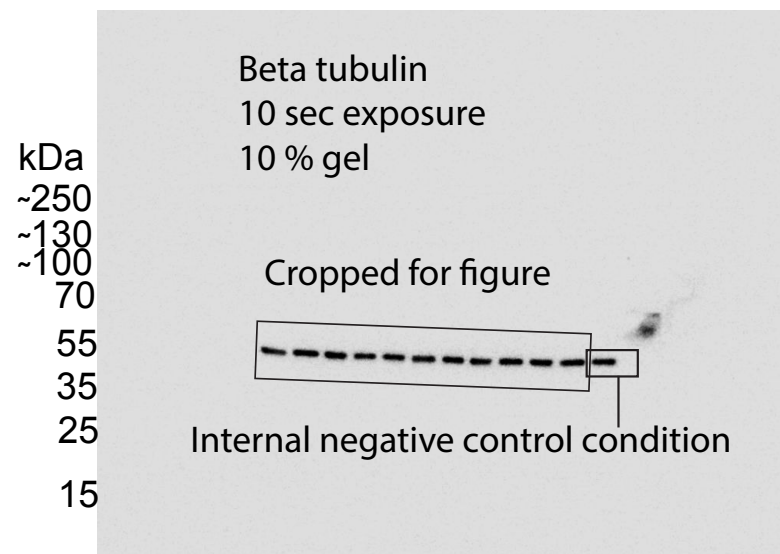

Supplement: Supplementary file 8 — Unprocessed western blots. [file 43587_2026_1101_MOESM8_ESM.pdf]

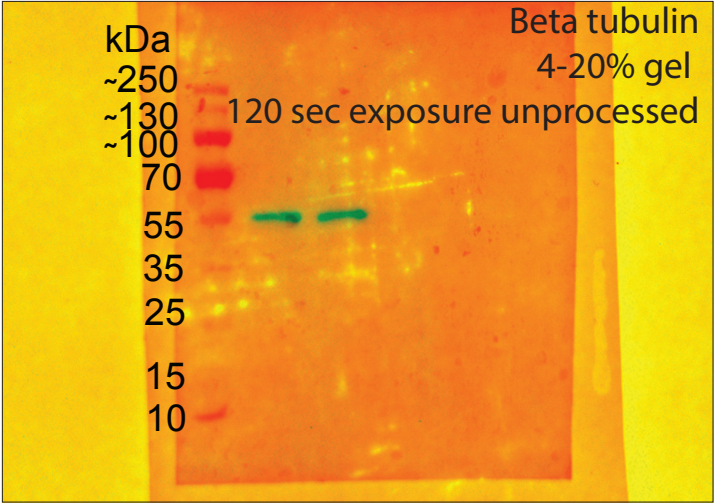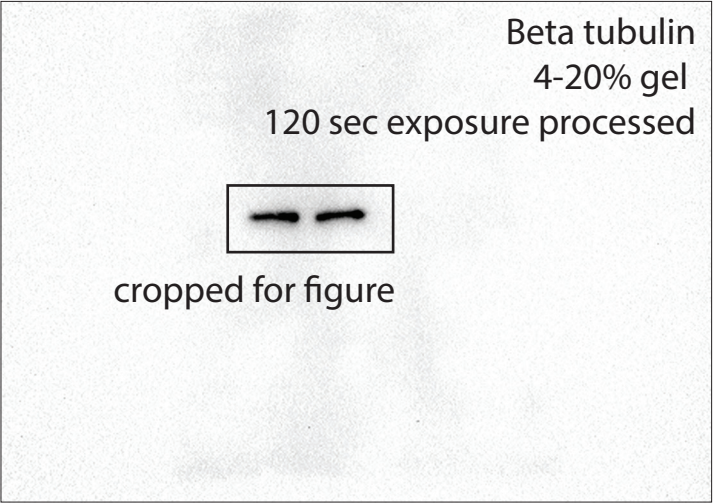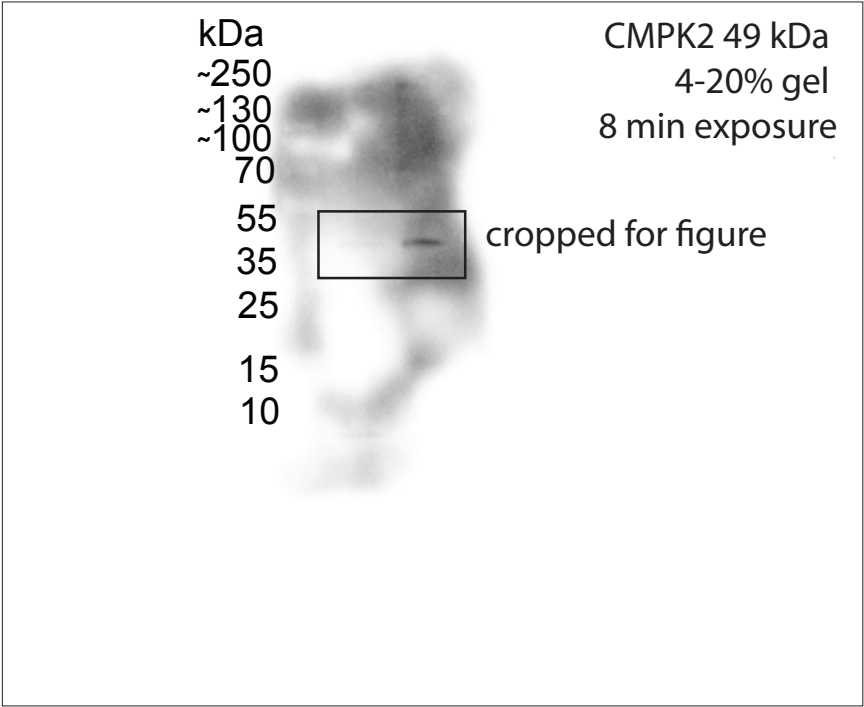

Supplement: Supplementary file 9 — Unprocessed western blots. [file 43587_2026_1101_MOESM9_ESM.pdf]

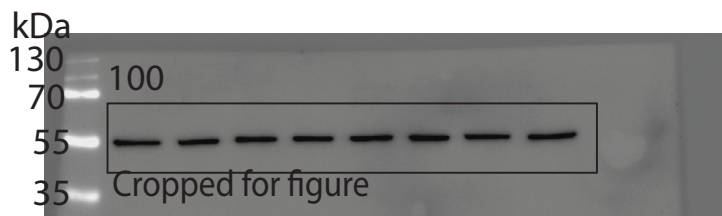

Beta Tubulin  
12% gel  
10 sec exposure

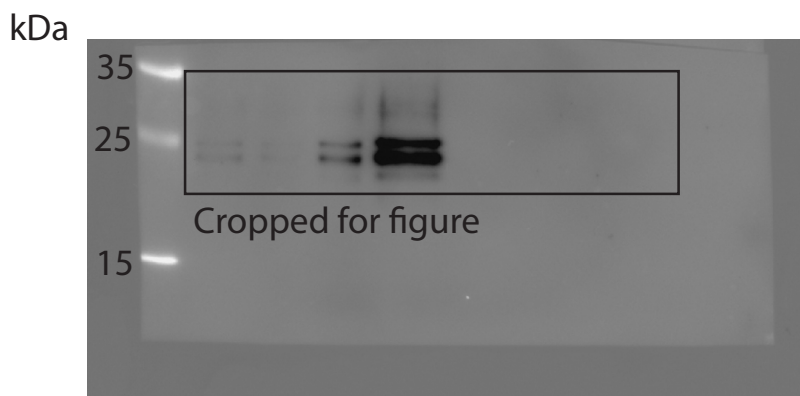

TREM2  
12% gel  
30 sec exposure

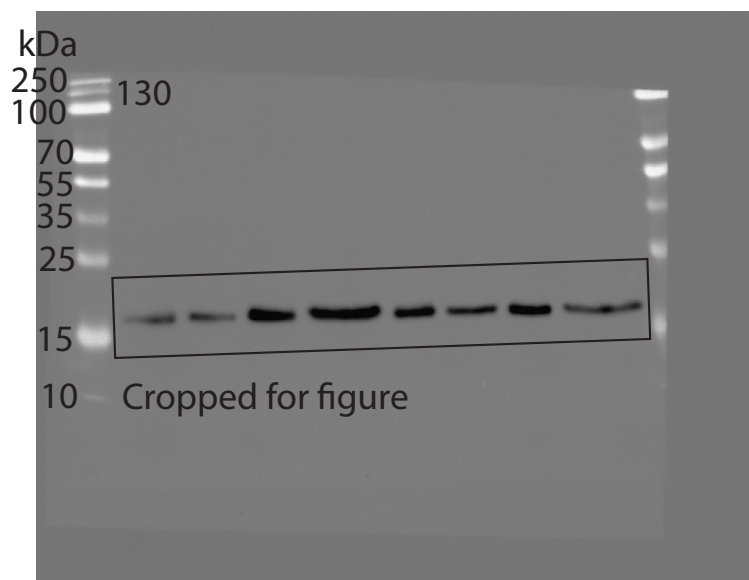

p21  
15% gel  
1 min exposure

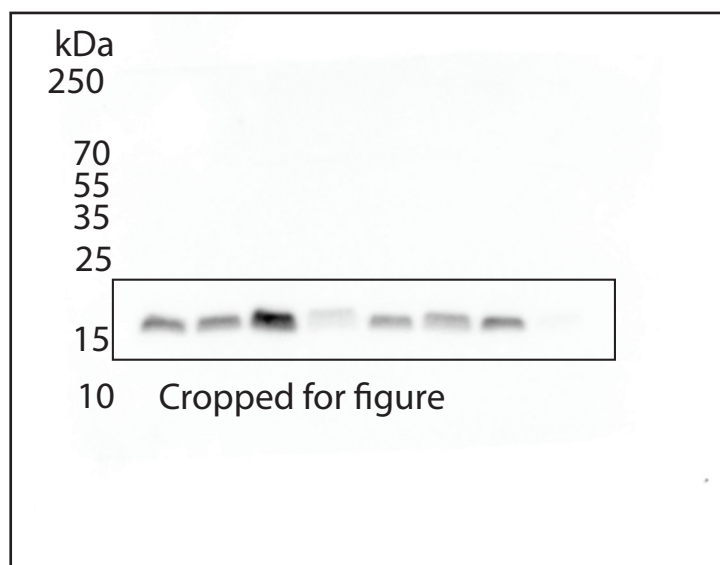

p16  
15% gel  
15 sec exposure

Supplement: Supplementary file 12 — Unprocessed western blots. [file 43587_2026_1101_MOESM12_ESM.pdf]

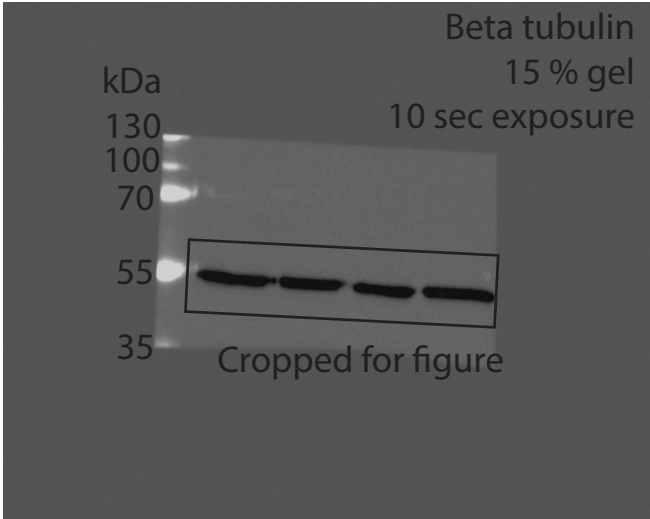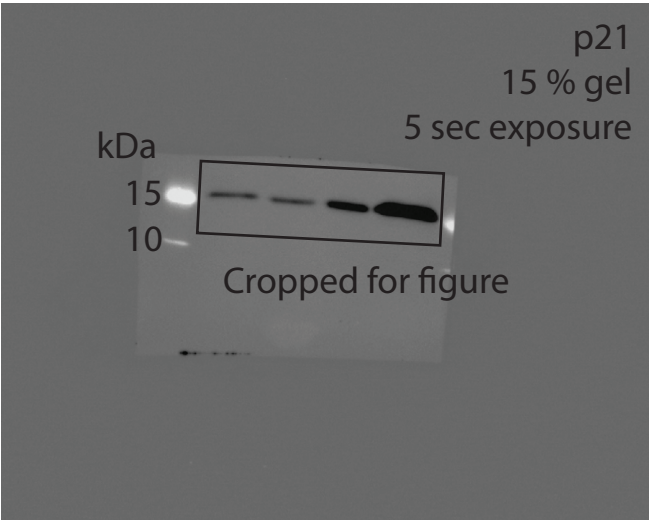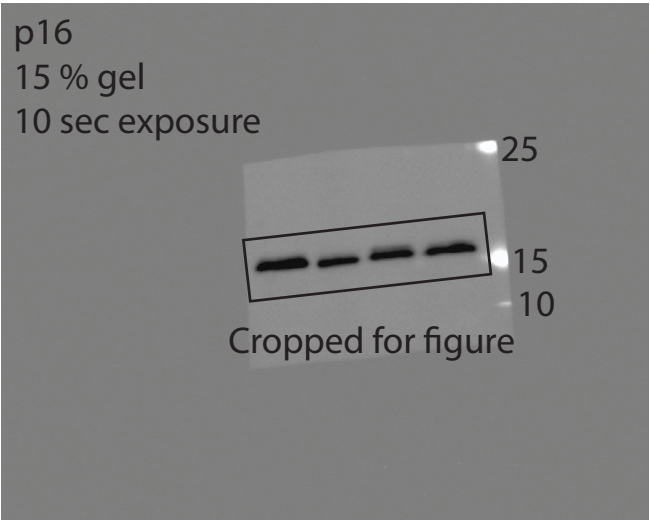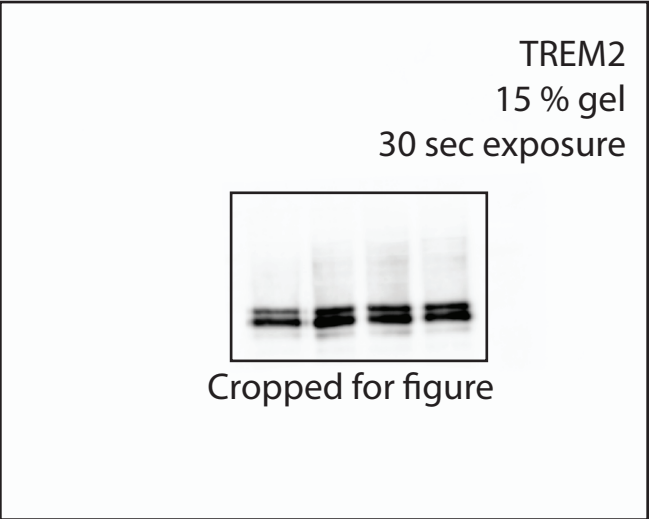

Supplement: Supplementary file 14 — Unprocessed western blots. [file 43587_2026_1101_MOESM14_ESM.pdf]

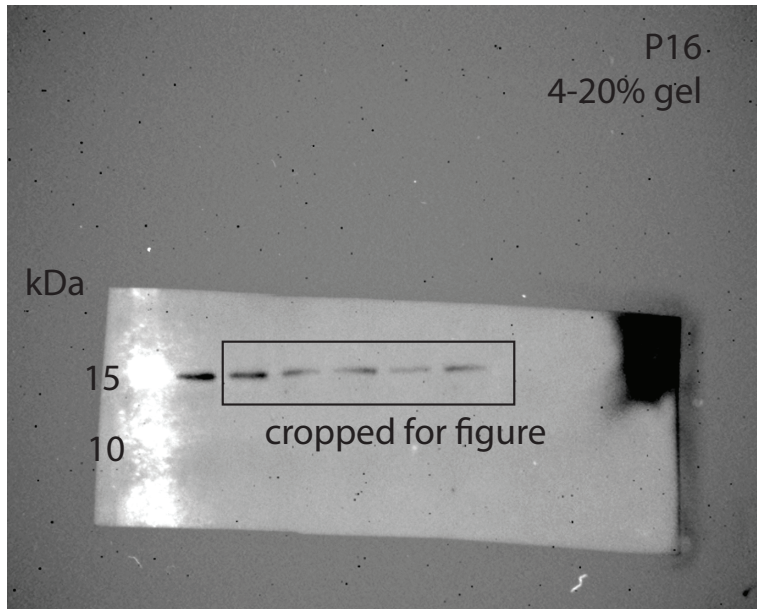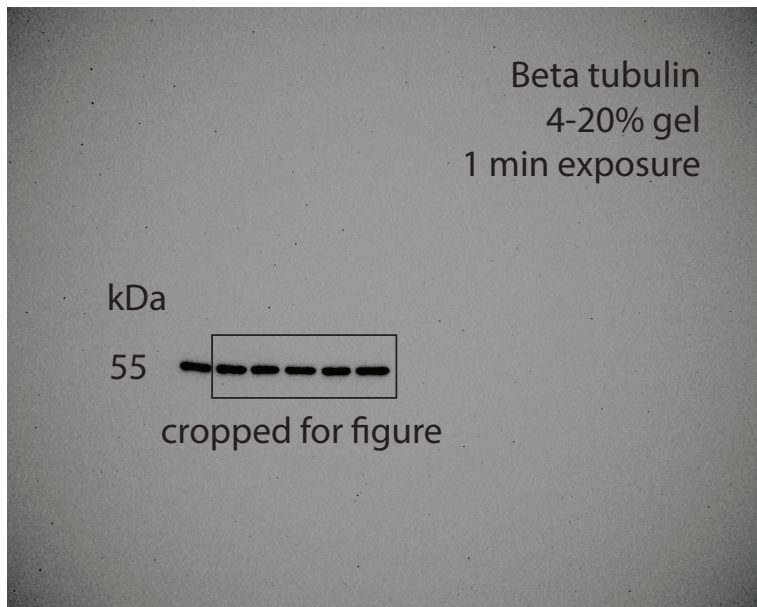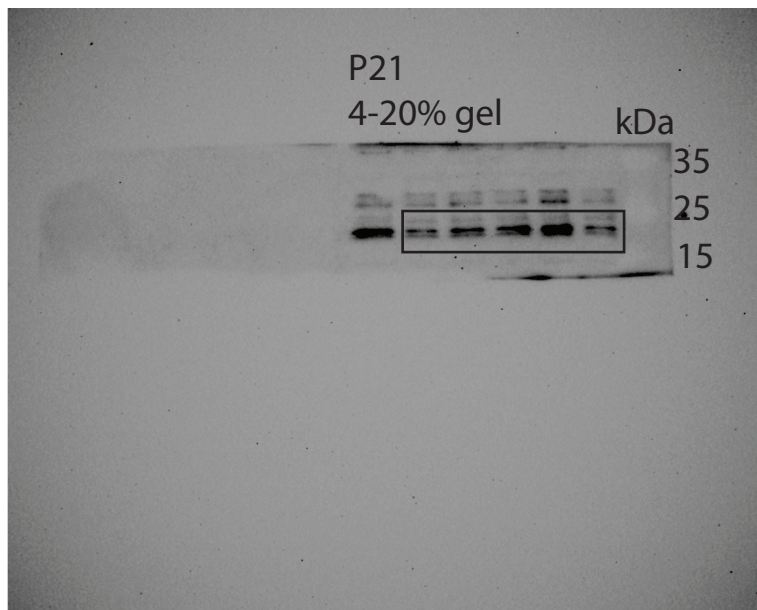

Supplement: Supplementary file 18 — Unprocessed western blots. [file 43587_2026_1101_MOESM18_ESM.pdf]
